# Association between the AUC_0-24_/MIC Ratio of Vancomycin and Its Clinical Effectiveness: A Systematic Review and Meta-Analysis

**DOI:** 10.1371/journal.pone.0146224

**Published:** 2016-01-05

**Authors:** Peng Men, Hui-Bo Li, Suo-Di Zhai, Rong-Sheng Zhao

**Affiliations:** 1 Department of Pharmacy, Peking University Third Hospital, Beijing, China; 2 Department of Pharmacy Administration and Clinical Pharmacy, School of Pharmaceutical Sciences, Peking University, Beijing, China; Ella Foundation, INDIA

## Abstract

**Background:**

A target AUC_0-24_/MIC ratio of 400 has been associated with its clinical success when treating *Staphylococcus aureus* infections but is not currently supported by state-of-the-art evidence-based research.

**Objective:**

This current systematic review aimed to evaluate the available evidence for the association between the AUC_0-24_/MIC ratio of vancomycin and its clinical effectiveness on hospitalized patients and to confirm the existing target value of 400.

**Methods:**

PubMed, Embase, Web of Sciences, the Cochrane Library and two Chinese literature databases (CNKI, CBM) were systematically searched. Manual searching was also applied. Both RCTs and observational studies comparing the clinical outcomes of high AUC_0-24_/MIC groups versus low AUC_0-24_/MIC groups were eligible. Two reviewers independently extracted the data. The primary outcomes were mortality and infection treatment failure. Risk ratios (RRs) with 95% confidence intervals (95%CIs) were calculated.

**Results:**

No RCTs were retrieved. Nine cohort studies were included in the meta-analysis. Mortality rates were significantly lower in high AUC_0-24_/MIC groups (RR = 0.47, 95%CI = 0.31–0.70, p<0.001). The rates of infection treatment failure were also significantly lower in high AUC/MIC groups and were consistent after correcting for heterogeneity (RR = 0.39, 95%CI = 0.28–0.55, p = 0.001). Subgroup analyses showed that results were consistent whether MIC values were determined by broth microdilution (BMD) method or Etest method. In studies using the BMD method, breakpoints of AUC_0-24_/MIC all fell within 85% to 115% of 400.

**Conclusions:**

This meta-analysis demonstrated that achieving a high AUC_0-24_/MIC of vancomycin could significantly decrease mortality rates by 53% and rates of infection treatment failure by 61%, with 400 being a reasonable target.

## Introduction

Vancomycin, a glycopeptide antibiotic, was developed and released in the 1950s for the treatment of aerobic gram-positive infections and has been widely used primarily in the treatment of methicillin-resistant *Staphylococcus aureus* (MRSA) infections[1]. In recent years, MRSA has spread globally, and the incidence of MRSA has shown a rising trend. Meanwhile, concerns have been raised about inadequate responses to vancomycin if the minimum inhibitory concentration (MIC) of the infecting organism lies at the upper end of the susceptible range[2]. Thus, there is a growing need for monitoring vancomycin levels, evaluating the relationship between its clinical effectiveness and pharmacokinetic parameters, and developing an individualized dosing regimen[3].

Vancomycin appears to follow time-dependent killing[4]. *In vitro* tests and limited human trials have shown that the ratio of the 24-hour area under the concentration-time curve and MIC (AUC_0-24_/ MIC) of vancomycin is the pharmacokinetic/pharmacodynamics (PK/PD) parameter that is most relevant to its clinical effectiveness in the treatment of *Staphylococcus aureus* infections[5,6]. An AUC_0-24_/MIC ratio higher than 400 is regarded as the target of clinical success. However, this conclusion was based on only one clinical observational study, which used the broth microdilution (BMD) method to test MIC, and had not been confirmedby performing a systematic review. Moreover, little was known regarding to what extent achieving high AUC_0-24_/MIC levels of vancomycin could enhance its clinical effectiveness. Thus, we performed a systematic review and meta-analysis to study these issues.

## Methods

### Search strategy

Previously published articles and conference abstracts (until Jan 16, 2014) reporting the association between the AUC_0-24_/MIC ratio of vancomycin and its clinical effectiveness were identified by computerized literature searches in PubMed, Embase, the Web of Science, the Cochrane Library and two Chinese literature databases (CNKI and CBM). Reference lists of the retrieved articles and supplemental materials were also examined manually to further identify potentially relevant studies. The search term was “vancomycin”. No restriction on language was applied. This study was a part of the development of Chinese practice guideline for therapeutic drug monitoring (TDM) of vancomycin launched by the Division of Therapeutic Drug Monitoring, Chinese Pharmacological Society.

### Selection criteria

The literature was divided into eight parts whose eligibility was assessed according to titles and abstracts by eight separate groups based on PICOs relating to the development of Chinese practice guideline for vancomycin TDM (See S1 File for details). Each group consisted of two independent assessors. Results of the assessment were converged as a whole.

Furthermore, two authors (PM and HBL) independently searched the literature and examined the relevant studies for further assessment of the data. Each reviewer was blinded to the other reviewer during the process of data extraction. In cases of disagreement between the two reviewers, a third author (SDZ) was consulted. Both randomized controlled trials (RCTs) and observational studies were eligible with the following inclusion criteria: 1) comparing clinical effectiveness of high and low AUC_0-24_/MIC levels of vancomycin monotherapy; 2) breakpoints of the AUC_0-24_/MIC ratio were reported; 3) patients were diagnosed with gram-positive infections with well-validated diagnostic criteria. Reviews, editorials, guidelines and case reports were excluded. We also contacted the authors for related information if data provided were insufficient.

### Data extraction and outcomes

All data were extracted independently by the two authors. Data extracted from the identified studies included the author (s), year of publication, country in which the study was conducted, study design, number of patients enrolled, population characteristics (type and etiology of infections), results of clinical outcomes and breakpoints of AUC_0-24_/MIC ratio. The outcomes of the review were all-cause mortality and infection treatment failure.

### Quality appraisal

Two authors independently assessed the quality of included studies. Discrepancies were resolved by discussion or through consultation with the third reviewer (SDZ). The potential risks of bias in RCTs were assessed according to the criteria developed using the Cochrane risk of bias tool. The quality of observational studies was assessed using the New Castle-Ottawa (NOS) scales.

### Statistical analysis

All statistical analyses were performed in STATA 12.0 (Stata Corp LP, College Station, TX, United States). Pooled risk ratios (RRs) and 95% confidence intervals (CIs) were calculated using the Mantel-Haenszel (M-H) fixed effects model if there was no evidence of significant heterogeneity for these outcomes, or random effects model if significant heterogeneity for these outcomes was present. Subgroup analyses were performed according to different methods for MIC determination. Heterogeneity among the studies was assessed using χ^2^ test and quantified using the Higgins I^2^[7]. To account for the low statistical power of the χ^2^ test for heterogeneity, P = 0.10 was considered not significant. If P<0.10, then a sensitivity analysis was performed to assess the validity of the outcomes. Publication bias was examined by Egger’s tests if there were at least five studies for each outcome[8]. The significance level was set at 0.05.

Methods for this systematic review were developed according to recommendations from the Preferred Reporting Items for Systematic Reviews and Meta-Analyses (PRISMA) checklists (See S2 File for details).

## Results

**Fig 1:**
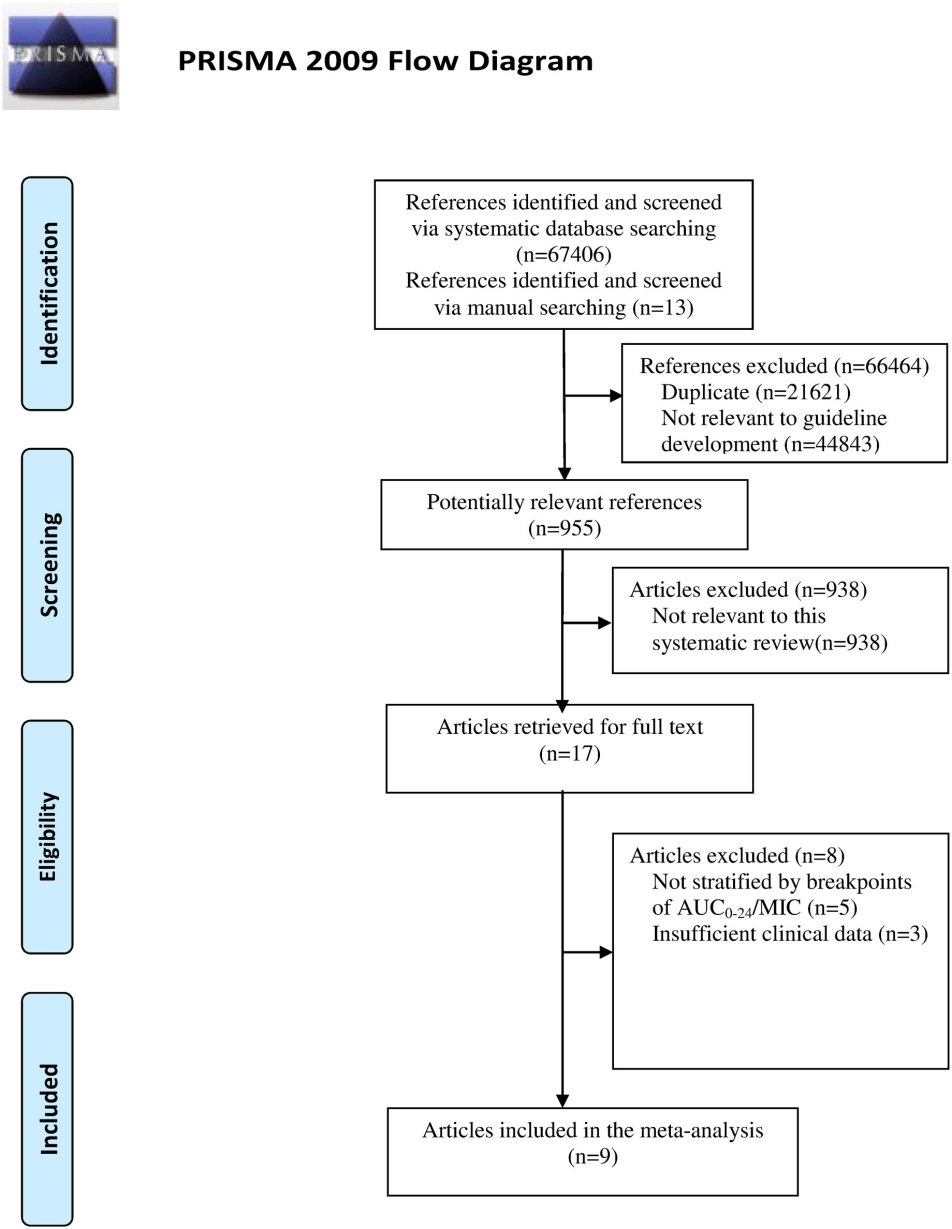
Flow chart depicting the selection process of studies included in the meta-analysis.

A total of 66464 were excluded from 67406 references after a review of titles: 21621 were duplicate and 44843 were not relevant to the guideline drafting. Next, we initially identified 942 potentially relevant studies using systematic database searching and 13 studies using manual searching. The full-text articles of the remaining 955 studies were evaluated. Another 938 studies were excluded because they were not relevant to this systematic review. Among the other 17 studies, 5 of the studies did not compare the clinical outcomes between the high and low AUC_0-24_/MIC ratio groups, 3 studies did not present sufficient clinical data. Nine studies were ultimately included in the meta-analysis. The flow chart of selection of studies and reasons for exclusion is presented in Fig 1.

### Study description

Studies included in our review were all cohort studies[9–17]. No RCTs were retrieved. These studies included a total of 916 patients. A summary description of the included studies is reported in Table 1. Of these studies, four studies were performed in the United States, two in Australia, and one each in Belgium, Canada, and the Republic of Korea.

**Table 1:** Main Characteristics of Studies Included in the Meta-analysis.

Reference (year)	Study design; country	Number of patients	Pathogens	Type of infections	Male,%	Mean age(SD)
Ampe et al.(2013) [9]	Prospective; Belgium	20	CoNS, MRSA, MSSA	Foreign body, osteomyelitis, septicaemia	70	65.6(12.6)
Brown et al.(2012)[10]	Retrospective; the United States	44	MRSA	Complicated bacteremia, infective endocarditis	50	54.8(16)
Gawronski et al.(2013)[11]	Retrospective; the United States	59	MRSA	Complicated bacteria, osteomyelitis	59	54(16)
Ghosh et al.(2014)[12]	Retrospective; Australia	127	MRSA	Abdominal sources, endocarditis, non-endocarditis vascular sources, pneumonia	68.5	64.6(NR)
Holmes et al.(2013)[13]	Prospective^a^; Australia	182	MRSA, MSSA	Endocarditis, osteoarticular, pneumonia, sepsis syndrome, skin and soft tissue	70	NR
Jung et al.(2014)[14]	Retrospective; the Republic of Korea	76	MRSA	Bone and joint, catheter-related, deep incisional/organ space, endocarditis, pneumonia, skin and soft tissue, surgical site	76.3	NR
Kullar et al.(2011)[15]	Retrospective; the United States	320	MRSA	Bone and joint, catheter-related, deep abscess, endocarditis, multiple sites, pneumonia, skin/wound	NR	54(NR)
Moise et al.(2000)[16]	Retrospective; the United States	53	MRSA, MSSA	Lower respiratory tract	61	69.1(15)
Zelenitsky et al.(2013)[17]	Retrospective; Canada	35	MRSA	Bloodstream, central nervous system, endocarditis, intra-abdominal, lower respiratory tract, skin/skin structure	62.9	61.9(15.2)

CoNS, coagulase-negative *Staphylococci*; MSSA, methicillin sensitive *Staphylococcus aureus*; NR, not reported; SD, standard difference.

^a^ Additional clinical data required for analysis were collected retrospectively using a detailed chart review.

### Quality of the included studies

Quality appraisal of the included cohort studies is shown in Table 2. Six studies completely accounted for the nine factors assessed. One study was adequate in eight of the nine factors, and two studies were adequate in seven of the nine factors.

**Table 2:** Quality Appraisal of Observational Studies.

References(year)	Quality indicators								
	1^a^	2^b^	3^c^	4^d^	5A^e^	5B^f^	6^g^	7^h^	8^i^
Ampe et al.(2013)	Yes	Yes	Yes	Yes	Yes	Yes	Yes	Yes	Yes
Brown et al.(2012)	Yes	Yes	Yes	Yes	Yes	Yes	Yes	Yes	Yes
Gawronski et al.(2013)	Yes	Yes	Yes	Yes	/	/	Yes	Yes	Yes
Ghosh et al.(2014)	Yes	Yes	Yes	Yes	Yes	Yes	Yes	Yes	Yes
Holmes et al.(2013)	Yes	Yes	Yes	Yes	No	Yes	Yes	Yes	Yes
Jung et al.(2014)	Yes	Yes	Yes	Yes	Yes	Yes	Yes	Yes	Yes
Kullar et al.(2011)	Yes	Yes	Yes	Yes	Yes	Yes	Yes	Yes	Yes
Moise et al.(2000)	Yes	Yes	Yes	Yes	Yes	Yes	Yes	Yes	Yes
Zelenitsky et al.(2013)	Yes	Yes	Yes	Yes	No	No	Yes	Yes	Yes

^a^ Indicates exposed cohort truly representative.

^b^ Non-exposed cohort drawn from the same community.

^c^ Ascertainment of exposure from a secure record.

^d^ Outcome of interest not present at start of study.

^e^ Cohorts comparable on basis of site and etiology of infections, or APACHEII Score.

^f^ Cohorts comparable on other factors.

^g^ Assessment of outcome of record linkage or independent blind assessment.

^h^ Follow-ups that were sufficient for outcomes to occur.

^i^ Complete accounting for cohorts.

### Outcomes

#### Mortality

**Fig 2:**
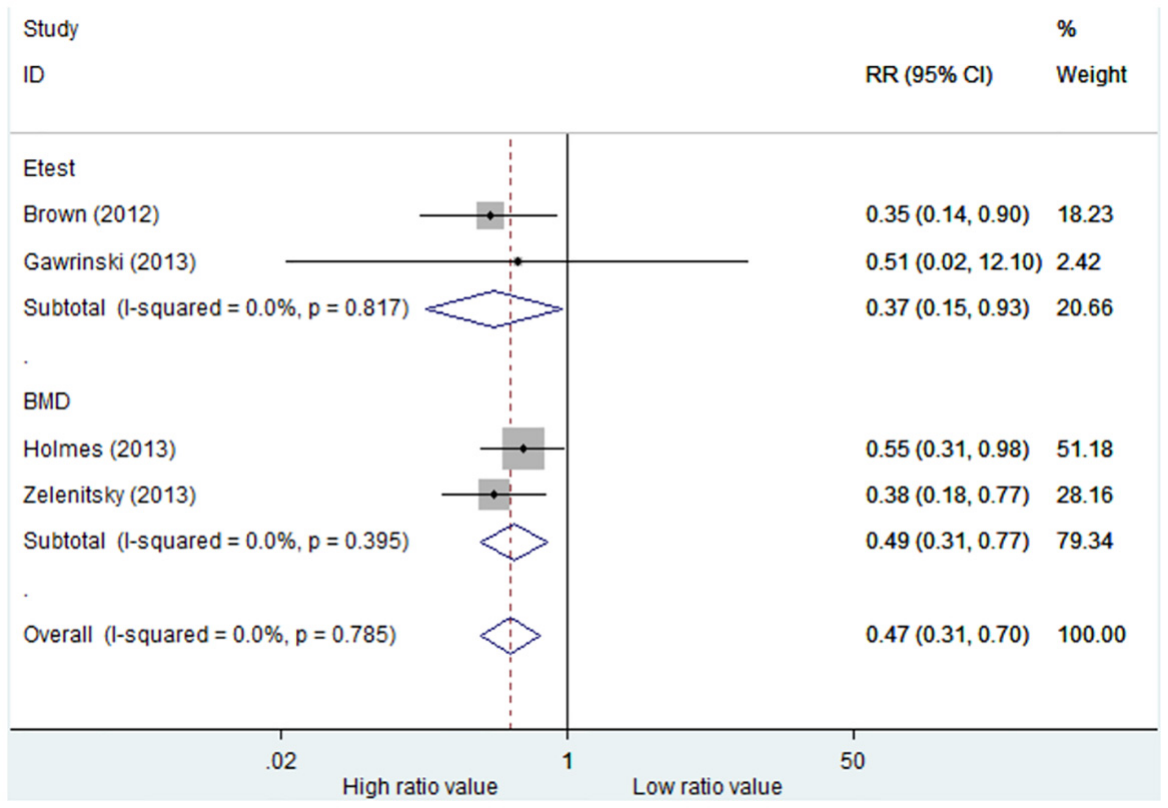
Risk ratios of all-cause mortality rates: high versus low AUC_0-24_/MIC ratio. Test of all-cause mortality rates for overall effect: Z = 3.72, P<0.001; test of all-cause mortality rates in Etest Study subgroup for overall effect: Z = 2.12, P = 0.034; test of all-cause mortality rates in BMD Study subgroup for overall effect: Z = 3.12, P = 0.002.

Four studies reported all-cause mortality. There was no significant heterogeneity among these studies (p = 0.785,I^2^ = 0.0%), so a fixed effects model was used (Fig 2). Compared with low AUC_0-24_/MIC groups, the high AUC_0-24_/MIC groups had significantly lower mortality rates (RR = 0.47, 95%CI = 0.31–0.70, p<0.001).

Among these studies, two studies applied the BMD method (referred to as the “BMD Study”), and the remaining studies applied the Etest method (referred to as the “Etest Study”) for MIC determination. Subgroup analyses showed that in both the BMD Study and Etest Study, high AUC_0-24_/MIC groups had significantly lower mortality rates (BMD: RR = 0.49, 95%CI = 0.31–0.77, p = 0.002; Etest: RR = 0.37, 95%CI = 0.15–0.93, p = 0.034). No significant heterogeneity was found (BMD: p = 0.395, I^2^ = 0.0% and Etest: p = 0.817,I^2^ = 0.0%).

#### Rate of infection treatment failure

**Fig 3:**
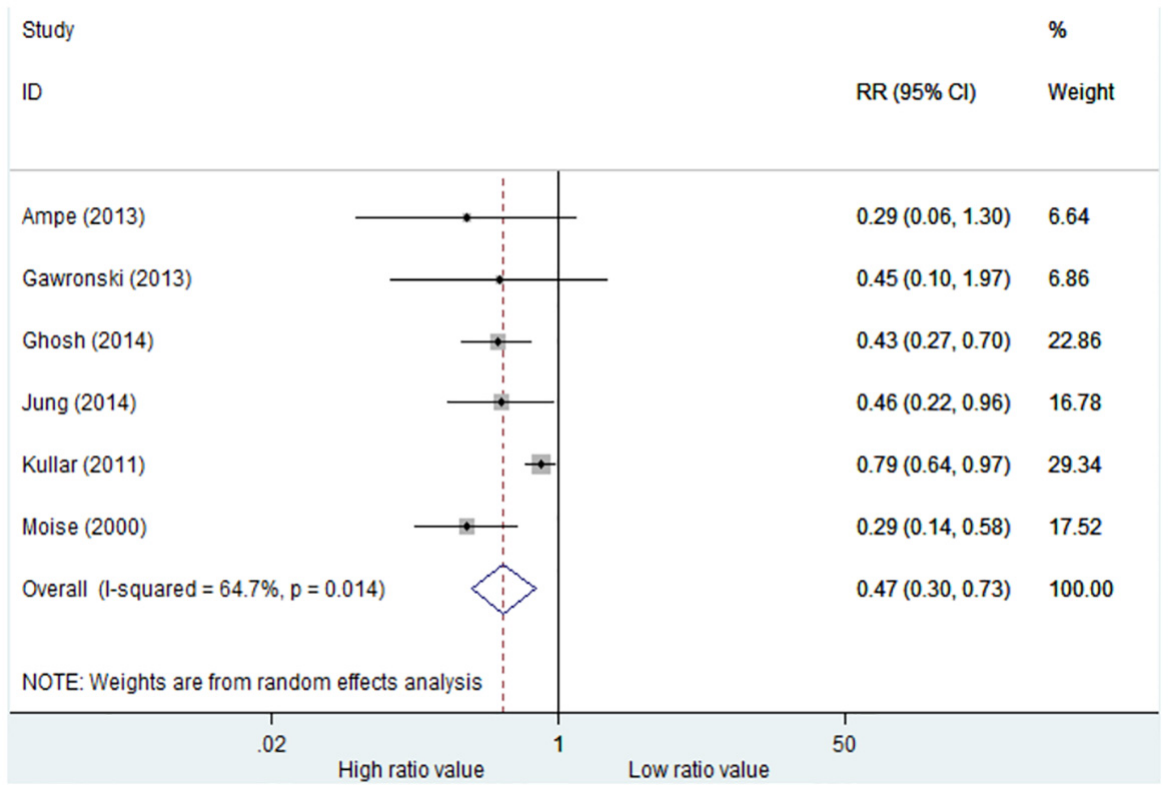
Risk ratios of rates of infection treatment failure: high versus low AUC_0-24_/MIC ratio. Test of rates of infection treatment failure for overall effect: Z = 3.34, P = 0.001.

Six studies reported rates of infection treatment failure. There was significant heterogeneity among these studies (p = 0.014, I^2^ = 64.7%), and thus we used a random-effect model (Fig 3). Compared with low AUC_0-24_/MIC groups, high AUC_0-24_/MIC groups had significantly lower rates of infection treatment failure (RR = 0.47, 95%CI = 0.30–0.73, p = 0.001). These six studies applied the BMD method to determine the MIC values.

#### Breakpoints of the AUC_0-24_/MIC ratio

Among the studies included in this systematic review, seven studies applied BMD method to determine the MIC values. All breakpoints reported from these studies fell within 85% to 115% of 400, ranging from 345 to 451 (See Table 3).

**Table 3:** Outcomes, breakpoints of AUC_0-24_/MIC ratio and MIC determination methods of studies included in the meta-analysis.

Reference(year)	Outcomes available in systematic review	All-cause mortality (deaths/total)		Infection treatment failure (failures/total)		Breakpoints of AUC_0-24_/MIC ratio	MIC determination methods
		Higher than breakpoint	Lower than breakpoint	Higher than breakpoint	Lower than breakpoint		
Ampe et al.(2013)	Infection treatment failure	/	/	2/14	3/6	451	BMD
Brown et al.(2012)	All-cause mortality	6/37	6/7	/	/	211	Etest
Gawronski et al.(2013)	All-cause mortality; Infection treatment failure	0/23	1/36	2/23	7/36	293	Etest
Ghosh et al.(2014)	Infection treatment failure	/	/	18/77	27/50	398	BMD
Holmes et al.(2013)	All-cause mortality	17/108	21/74	/	/	373	BMD
Jung et al.(2014)	Infection treatment failure	/	/	10/52	10/24	398.5	BMD
Kullar et al.(2011)	Infection treatment failure	/	/	107/221	61/99	421	BMD
Moise et al.(2000)	Infection treatment failure	/	/	7/32	16/21	345	BMD
Zelenitsky et al.(2013)	All-cause mortality	6/20	12/15	/	/	451	BMD

### Heterogeneity and publication bias

**Fig 4:**
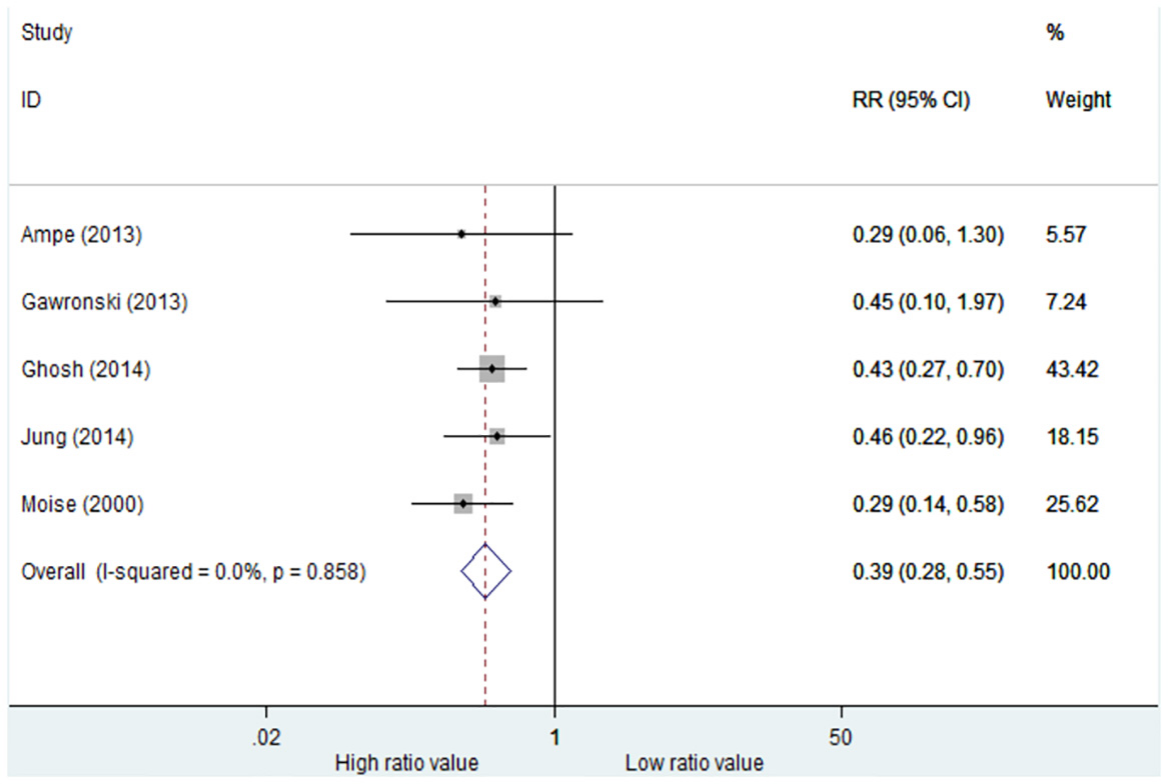
Risk ratios of rates of infection treatment failure: high versus low AUC_0-24_/MIC ratio (Kullar’s study was excluded). Test of rates of infection treatment failure for overall effect: Z = 5.55, P<0.001.

Because there was significant heterogeneity among the studies reporting rates of infection treatment failure, we performed a sensitivity analysis (See S1 Fig), which showed that Kullar’s study had a significant effect on the meta-analysis result. Heterogeneity was not significant after we excluded that study (I^2^ = 0.0%, p = 0.858) and results were consistent (See Fig 4, RR = 0.39, 95%CI = 0.28–0.55, p = 0.001).

For rates of infection treatment failure, the amount of studies was enough to perform the Egger’s test for publication bias. No significant result was observed (P = 0.706).

## Discussion

Our systematic review and meta-analysis demonstrated that high values of AUC_0-24_/MIC had significant advantages compared to low values in terms of all-cause mortality rates (reduced by 53%) and rates of infection treatment failure (reduced by 53%, coming to 61% after correcting for heterogeneity) by reaching the breakpoint of 400. To the best of our knowledge, this report describes the first systematic review to support this hypothesis. Our research has raised the grade of evidence concerning this issue, which may be beneficial for clinicians, clinical pharmacists and future therapeutic drug monitoring guideline writers.

Kullar’s study was excluded from meta-analysis because of its significant effect on heterogeneity. The possible reason of the effect was that patients among the cohort had relatively higher baseline APACHE-II scores.

Importantly, the apparent values of AUC_0-24_/ MIC ratio vary depending on different MIC determination methods. Commonly applied methods include the BMD and Etest methods. Latter results are consistently 0.5 to 1.5 times higher than the former results after log2 conversion[18,19]. A target value of 400 recommended by the American guideline was based on results obtained from BMD method[6]. Among the studies included in this meta-analysis, 7 studies applied BMD method. Breakpoints of the AUC_0-24_/MIC ratio were entirely within the range of 85–115% of 400. In addition, because the breakpoints were calculated using a fine classification and regression tree (CART) algorithm and a margin of error may exists, there will be slight differences. Different infection sites and pathogens may also cause the variations, although apparent associations were difficult to be found based on included studies. Our study had confirmed the target AUC_0-24_/MIC value of 400.

Two studies reported 211 and 293 as breakpoints respectively. The Etest method was applied for MIC determination, which probably accounting for the lower values. Holmes *et al*.[13] had performed a linear regression analysis and found that the target of 400 (based on BMD method) was equivalent to 226 based on the Etest method, which was very close to 211. This provided evidence for our analysis.

Pharmacokinetic and pharmacodynamics reviews have recommended the AUC_0-24_/MIC ratio as the preferred parameter partly based on data obtained from animal models, *in vitro* studies and limited human studies[20–23]. Moise-Broder *et al*. explored the use of AUC_0-24_/MIC ratio in predicting clinical and microbiological success in the treatment of ventilator-associated *S*. *aureus* pneumonia. An AUC_0-24_/MIC ratio of ≥ 400 was advocated as a target to achieve clinical success with vancomycin[6]. On the basis of these studies, the vancomycin TDM guideline (published in 2009) developed by American Society of Hospital-System Pharmacists recommended 400 as a clinically target, which requiring further support from solid evidence-based research.

However, there are some deficiencies in this study. First, a relatively small number of studies were included, which were all observational ones. Second, there was significant heterogeneity among the six studies, which reported the rate of infection treatment failure. After we found and excluded the questionable study, the problem was resolved and meta-analysis results were consistent.

This study focused on the relevant predictors of vancomycin clinical effectiveness. At the present, researches on the association between vancomycin AUC_0-24_/ MIC and safety are limited. Some studies showed that high ratio values may increase the incidence of nephrotoxicity[18]. Neely *et al*.[24] discovered that when the AUC_0-24_/ MIC ratio was higher than 700, the incidence of nephrotoxicity was significantly increased, based on a population model. Lodise *et al*. [25] found that in 27 patients whose AUC_0-24_/ MIC values were equal to or higher than 1300, 26% had developed nephrotoxicity. Additional studies on nephrotoxicity are needed in the future.

In conclusion, this report describes the first systematic review indicating that achieving a high AUC_0-24_/MIC ratio of vancomycin could significantly decrease mortality rates by 53% and rates of infection treatment failure by 61%, with 400 being a reasonable target.

## Supporting Information

S1 Fig Sensitivity analysis of the studies reporting the rate of infection treatment failure. (TIF)

S1 File A protocol of Chinese practice guideline for therapeutic drug monitoring of vancomycin (Including details of the first step of literatures selection). (PDF)

S2 File PRISMA 2009 Checklist. (DOC)

S3 File List of full-text excluded articles and reasons for exclusion. (DOCX)
