# Supplementary figures and images for: Association between the AUC0-24/MIC Ratio of Vancomycin and Its Clinical Effectiveness: A Systematic Review and Meta-Analysis
Source: PLoS One. 2016 Jan 5;11(1):e0146224. doi: 10.1371/journal.pone.0146224 (PMC4701440; doi:10.1371/journal.pone.0146224)

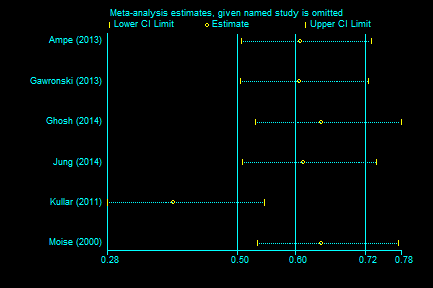

Supplement: S1 Fig — (TIF) [file pone.0146224.s001.tif]
