# Supplementary material for: Association between the AUC0-24/MIC Ratio of Vancomycin and Its Clinical Effectiveness: A Systematic Review and Meta-Analysis
Source: PLoS One. 2016 Jan 5;11(1):e0146224. doi: 10.1371/journal.pone.0146224 (PMC4701440; doi:10.1371/journal.pone.0146224)
Supplement: S1 File — (PDF) [file pone.0146224.s002.pdf]

A Protocol of Chinese Practice Guideline for Therapeutic Drug Monitoring of Vancomycin

Zhi-Kang Ye[1]， Ken Chen[1, 2]， Yao-Long Chen[3, 4]， Suo-Di Zhai[1*]

[1]Department of Pharmacy, Peking University Third Hospital, Beijing, China

[2]Department of Pharmacy Administration and Clinical Pharmacy, School of Pharmaceutical Sciences, Peking University Health Science Center, Beijing, China

[3]Evidence-Based Medicine Center, School of Basic Medical Sciences, Lanzhou University, Gansu, China

[4]Chinese GRADE Center, Gansu, China

Corresponding author: Suo-Di Zhai. No.49, Huayuan North Road, Haidian District, Beijing, China. 100191

Email: zhaisuodi@163.com Telephone number: 13401169143

Key words: Vancomycin, Therapeutic drug monitoring, Guideline, GRADE

Word Count: 2910

Abstract:

Background: Vancomycin is currently the first-line treatment for infections caused by methicillin-resistant Staphylococcus aureus (MRSA).Monitoring vancomycin serum concentrations significantly increases the rate of clinical efficacy and decreases the rate of nephrotoxicity in patients treated with vancomycin. Both America and Japan recently published guidelines for vancomycin therapeutic drug monitoring (TDM)in 2009 and 2013, respectively. However, not all of their recommendations were applicable to Chinese patients because of population differences. The two guidelines did not use the same rating system, neither of which was the new and popular rating system—Grade of Recommendations Assessment, Development and Evaluation (GRADE). Only a few hospitals in China conduct vancomycin TDM, and where this is conducted, the standards are inconsistent, with just under 50% of the monitored trough serum vancomycin concentrations under 10μg/ml.

Methods: We followed the new definition of guideline from Institute of Medicine (IOM), adhered closely to the six domains of Appraisal of Guidelines for Research & Evaluation II(AGREEII), and made recommendations based on systematic reviews. As far as we know, we have develop the first evidenced-based guideline for vancomycin TDM under the framework of GRADE.

Results: We stablished a Guideline Steering Group and a Guideline Development Group, formulated 12 questions in the form of PICOs (Population, Intervention, Comparison, Outcome) and completed a literature search.

Conclusion: We aim to develop the first evidenced-based guideline for vancomycin TDM for Chinese patients, which will set the standards for vancomycin TDM in China.

Registration: The guideline is registered in the Global Practice Guidelines Registry Platform and the registration number is GR-20140103.

Key words: Vancomycin, Therapeutic drug monitoring, Guideline, GRADE

Background:

Vancomycin is currently the first-line treatment for infections caused by methicillin-resistant Staphylococcus aureus (MRSA)[1]. One study confirmed that vancomycin therapeutic drug monitoring (TDM) significantly increases the rate of clinical efficacy and decreases the rate of nephrotoxicity in patients treated with vancomycin[2]. Both America and Japan recently published guidelines for vancomycin TDM in 2009 and 2013, respectively, which recommended that vancomycin serum trough concentrations should be elevated from 5-10 μg/ml to 10-20 μg/ml, and that for serious or complicated infections, the vancomycin serum trough concentrations should be 15-20 μg/ml [3, 4].

A literature search found that few hospitals in China conduct vancomycin TDM. Of the hospitals that do conduct vancomycin TDM, only 19%adopted 10-20 μg/ml as their vancomycin target serum trough concentrations; just under 50% of patients' serum trough concentrations were below 10 μg/ml; 4% of hospitals did not adopt a vancomycin loading dose administration; and 57% of hospitals had a delayed timing of monitoring[5]. A study evaluated twelve vancomycin TDM guidelines using Appraisal of Guidelines for Research & Evaluation II (AGREE II).The result showed that the overall quality of guidelines for vancomycin TDM was moderate; the highest score was recorded in the domain of clarity of presentation, and the lowest score was recorded in the domain of rigor of development and stakeholder involvement. The specific recommendations of these guidelines were moderately consistent. Guideline developers should adhere more closely to AGREEII when developing or updating guidelines[6].

Although American and Japanese guidelines for vancomycin TDM have been published, there are several reasons why it is necessary to develop a Chinese guideline for vancomycin TDM. First, the American and Japanese recommendations are not applicable to Chinese patients because of differences between populations. For example, the vancomycin minimum inhibitory concentration (MIC) of MRSA is different indifferent states and countries[7]. In China, between 2006 and 2011, the vancomycin mean MIC of MRSA changed from 0.906 mg/L to 1.040 mg/L; in 2011, the MIC_{50} and MIC_{90} were 1 mg/L and 1.5 mg/L, respectively[8]. However, the results of the 2008 Japanese nationwide surveillance of the antibacterial susceptibility of respiratory pathogens showed that the MIC_{50} and $MIC_{90}$for vancomycin were 1 mg/L and 2 mg/L, respectively[9]. In addition, according to the American laboratory bacterial

resistance surveillance network, 16% of the MRSA isolates had a vancomycin MIC above 2 mg/L [10].Second, the American vancomycin TDM guideline has not been widely adopted by Chinese hospitals. Despite the publication of the American guidelines, 60% of Chinese hospitals have adopted 5-10 μg/ml as their vancomycin target serum trough concentrations; 26% monitor vancomycin peak concentrations, and44% of hospitals have a delayed timing of monitoring[5]. Third, clinical practice guidelines were defined as *statements that include recommendations intended to optimize patient care which are informed by a systematic review of evidence and an assessment of the benefits and harms of alternative care options by Institute of Medicine (IOM)*[11]. However, both the American and the Japanese guidelines for vancomycin TDM failed to meet the IOM requirements. The American and Japanese recommendations were not based on systematic reviews, economic evaluations were not conducted and patients' values and preferences were not considered. Finally the rating systems of these two guidelines were also not popular and up-to-date. We intend to develop an evidence-based guideline for vancomycin TDM in the framework of Grade of Recommendations Assessment, Development and Evaluation (GRADE).

We aim to develop a practical, applicable and updated vancomycin TDM guideline for Chinese patients, which provide evidence-based recommendations for vancomycin TDM to clinicians, pharmacists and nurses.

Methods:

Principle:

We followed the new definition of guideline form IOM and adhered closely to the six domains of AGREEII[12]. We referred to the World Health Organization handbook for guideline development[13]. We registered the guideline in Global Practice Guidelines Registry Platform and the registration number is GR2014050018[14, 15].

Guideline Development Institutions, Guideline Target Users and Target Population

The guideline was launched at the Chinese Third Annual Conference of TDM in Shanghai by the Peking University TDM and Clinical Toxicology Center and the Division of Therapeutic Drug Monitoring, Chinese Pharmacological Society in July 2013.Methodological support was provided by the Chinese GRADE Center. The title of the guideline was "Chinese Guideline for Therapeutic Drug Monitoring of Vancomycin", which was preliminarily developed by the Guideline Development

Group and approved by the Guideline Steering Group. The end-users of the guideline are physicians, pharmacists and nurses; the target population is patients treated with vancomycin. The content of the guideline is vancomycin TDM.

Guideline Development Group and Guideline Steering Group

The Guideline Development Group and the Guideline Steering Group were established in July 2013. To ensure fair representation by gender and region, the Guideline Development Group consists of 30 members from multiple fields of subjects as follows: 17 clinical pharmacists, 2 respiratory physicians, 1 infectious disease physician, 2 evidence-based medical experts, 2 medical laboratory scientists, 2 microbiologists, 1 pharmacologist, 1 pharmaco-economist, 1 pediatric physician and 1 nurse. The mission of Guideline Development Group is as follows:

1. To define the scope of the guideline and draft the PICO and to choose and rate the outcomes
2. To perform a literature search and complete systematic reviews
3. To grade the quality of the evidence
4. To investigate the patients' views and preferences
5. To draft preliminary recommendations
6. To write the draft guideline
7. To publish and promote the guideline

The Guideline Steering Group consists of 8 members, including 1 pharmacologist, 1 evidence-based medical expert, 3 clinical pharmacists, 2 respiratory physicians and 1 infectious disease physicians. The mission of Guideline Steering Group is as follows:

1. To approve the PICOs
2. To supervise the literature search and systematic reviews
3. To check the grade of the evidence
4. To draft the final recommendations using a modified Delphi approach
5. To approve the publication of the guideline

Declaration of Interests and Funding Support

Statement of Interest: Members of the Guideline Steering Group and the Guideline Development Group are required to complete the declaration of interest forms before attending the guideline meetings to judge their potential conflicts of interest.

Formulating Questions and Choosing Outcomes

After its proposal by the Guideline Development Group and approval by the Guideline Steering Group, we finalized the PICO. The Guideline Development Group chose the outcomes and rated them by their importance. The scores of the outcomes ranged from 1 to 9;on this scale, 7-9 is considered critical, 4-6 is important and 1-3 is not important. For our guideline, the mortality rates, the treatment efficacy rates (the rate of clinical efficacy, treatment failure or treatment success) and the rate of nephrotoxicity are considered critical; the cost-effectiveness and the proportion of the vancomycin target concentrations are reached are considered important; and the pharmacokinetic parameters (half-life, volume of distribution, clearance, area under curve (AUC), the trough concentrations, the length of hospital stay, the length of vancomycin therapy and the microbiological eradication rates are considered not important.

We formulated the following12Questions and associated PICOs:

1. Is TDM required for patients treated with vancomycin?

P: All patients treated with vancomycin

I: Patient who received TDM

C: Patient who did not receive TDM

O: The rate of clinical efficacy, rate of nephrotoxicity, mortality rates, length of vancomycin therapy, length of hospital stay.

2. Economic evaluations of vancomycin TDM.

P: All patients treated with vancomycin

I: Patient who received TDM

C: Patient who did not receive TDM

O: Economic evaluation measures, such as cost-effectiveness, return on investment, etc.

3. Which groups of patients benefit more from vancomycin TDM?

E: Special groups patients treated with vancomycin

P: All patients treated with vancomycin

O: The rate of treatment failure, rate of nephrotoxicity, mortality rates, proportion of the target trough concentrations that are reached, pharmacokinetic parameters (half-life, volume of distribution, clearance, AUC）， trough concentrations

4. The relationship between the AUC/MIC and vancomycin clinical outcomes.

P： All patients treated with vancomycin

I：Patients with higher AUC/MIC

C：Patients with lower AUC/MIC

O: Mortality rates, rate of treatment failure, rate of nephrotoxicity

5. What is the relationship between vancomycin trough concentrations and nephrotoxicity

P：All patients treated with vancomycin

I：Patients with higher vancomycin trough concentrations (stratified by defined levels)

C：Patients with lower vancomycin trough concentrations (stratified by defined levels)

O: The rate of nephrotoxicity

6. What is the relationship between vancomycin peak concentrations and clinical outcomes

P：All patients treated with vancomycin

I：Patients with higher vancomycin peak concentrations

C：Patients with lower vancomycin peak concentrations

O: Mortality rates, rate of treatment failure, rate of nephrotoxicity

7. When should the monitoring of vancomycin trough concentrations begin?

P: All patients treated with vancomycin

I: Patients in whom Cmin is measured before the Nth drug dose

C: Patients in whom Cmin is measured before the N+1/+2/…th drug dose

O: Mortality rates, rate of treatment failure, rate of nephrotoxicity, changes in the Cmin between initial and steady state.

8. What is the target vancomycin trough concentration for Chinese patients?

P: All patients treated with vancomycin

I: Patients with higher vancomycin trough concentrations (stratified by 10, 15, 20 mg/Liter)

C: Patients with lower vancomycin trough concentration (stratified by 10, 15, 20 mg/Liter)

O: Mortality rates, rate of treatment failure, rate of nephrotoxicity

9. Is an initial loading dose of vancomycin required at first administration?

P: All patients treated with vancomycin

I: Patients who receive an initial loading dose

C: Patients who do not receive an initial loading dose

O: Mortality rates, rate of clinical efficacy, rate of nephrotoxicity, proportion of

patients in which target trough concentrations are reached

10. Which of the following is better: intermittent infusion or a continuous infusion of vancomycin?

P: All patients treated with vancomycin

I: Patients who receive intermittent infusion of vancomycin

C: Patients who receive continuous infusion of vancomycin

O: Rate of nephrotoxicity, mortality rates, rate of treatment failure, rate of adverse drug reactions.

11. What is the relationship between vancomycin initial dosing regimen and clinical outcomes?

P: All patients treated with vancomycin

I: Patients in whom an initial dosing regimen is developed using patient-tailored population pharmacokinetics

C: Patients in whom an initial dosing regimen is developed without using patient-tailored population pharmacokinetics

O: Mortality rates, rate of treatment success, rate of nephrotoxicity, proportion of the target trough concentrations achieved, microbiological eradication rates

12. How should the dosing regimen be adjusted?

P: All patients treated with vancomycin

I: Patients in whom the dosing regimen is adjusted using population pharmacokinetics

C: Patients in whom the dosing regimen is adjusted without using population pharmacokinetics

O: Mortality rates, rate of treatment success, rate of nephrotoxicity, proportion of reaching target trough concentrations, microbiological eradication rates

Evidence Retrieval and Synthesis

Databases Searched:

We systematically searched the literature (until January 16, 2014) in PubMed, Embase, the Cochrane Library and three Chinese literature databases (CNKI, CBM and WanFang).

Search Term:

Our search terms were the combination of text free terms and Medical Subject Headings (MeSH) terms as “Vancomycin”. We also used the search terms “human” in PubMed and “human and (case report or clinical article or clinical protocol or clinical trial or cohort analysis or comparative study or controlled clinical trial or controlled

study or major clinical study or medical record review or meta-analysis or multicenter study or observational study or outcomes research or practice guideline or prospective study or randomized controlled trial or retrospective study or systematic review) and (article or article in press or conference paper or conference review or review or short survey) and (bacteremia or bacterial endocarditis or bacterial infection or bacterial meningitis or catheter infection or diarrhea or endophthalmitis or fever or hospital infection or infection or kidney failure or methicillin resistant staphylococcus aureus infection or nephrotoxicity or neutropenia or osteomyelitis or pneumonia or postoperative infection or sepsis or side effect or skin infection or staphylococcus infection or urinary tract infection)" in Embase.

Pilot search:

To ensure the consistency of the literature selecting standards, the authors of the systematic reviews conducted a pre-test. We randomly selected 64 bibliographical references for the pre-test. By summarizing the results of our literature selection and discussing the inconsistencies, all of the authors had a definite understanding of the inclusion and exclusion criteria.

Results of Literature Selection:

We identified 67406 studies, of which 21621 were duplicate articles. After excluding 44842 studies that were not relevant using the titles and abstracts, we included 943 studies for full-text reading. 16 pharmacists, which were divided to 8 groups, fulfilled the literature selection and reading.

Evidence Assessment:

We will use the GRADE approach to assess the quality of a body of evidence and to develop and report recommendations. According to the GRADE approach, the quality of evidence is categorized as high, moderate, low and very low. Randomized controlled trials are categorized as high-quality evidence and observational studies as low-quality evidence. We will conduct the assessment of evidence across studies on an outcome-by-outcome basis. The guideline methodologists will be responsible for quality assessment, drafting the evidence summaries and presenting these summaries at the Guideline Development Group meeting.

Patients' Values and Preferences

We will investigate patients' values and preferences towards vancomycin TDM. The results of the investigation will be analyzed and considered by the Guideline Steering Group and the Guideline Development Group when the recommendations

are formulated.

Developing Recommendations:

After completion of the GRADE evidence profile, the Guideline Development Group will draft preliminary recommendations based on the quality of the evidence, the balance between the benefits and harms, the patients' values and preferences and the health resources. The Guideline Development Group will develop the draft recommendations through 2-4 rounds of the Delphi process and submit the draft recommendations to the Guideline Steering Group for a final approval. We will refer to GRADE Grid to reach our consensus[16]. 5 choices of "Strong recommendation" "Weak recommendation" "Unclear recommendation" "Weak disrecommendation" and "Strong disrecommendation" will be used for each draft items on the questionnaire, respectively. As for each item, if more than 50% of the experts vote for one choice except the "unclear" one, or if more than 70% of the experts vote for the 2 choices on the same side, this will mean that the consensus of the item is reached. Otherwise, the item is controversial and needs one more rounds of the Delphi process.

Peer Review of Guideline:

The guideline will be submitted to approximately ten external experts for peer review. The Guideline Development Group will record the review process and the responses to the reviews.

The Report, Publication and Update of the Guideline:

The guideline will be published using the format recommended by the Essential Reporting Items for Practice Guidelines in Healthcare(RIGHT) working group[17]. It is estimated that the full text will be published in early 2015. Our guideline will be translated into English and Chinese and published in relevant English and Chinese journals. We plan to update the guideline within 5 years.

Promotion, Implementation and Evaluation of the Guideline:

After the guideline is published, it will be promoted by the Division of Hospital Pharmacy, Chinese Pharmaceutical Association and the Division of Therapeutic Drug Monitoring, Chinese Pharmacological Society in the following ways: 1) the guideline will be presented at conferences relating to TDM or infectious diseases for 3 years; 2) a learning session for the guideline will be organized for physicians, pharmacists and nurses in China; 3)members of the Guideline Steering Group and the Guideline Development Group will write journal articles related to the guideline; and 4)the Chinese version of the guideline will be placed on popular native medical websites.

Research will be conducted to evaluate the impact of the guideline on the vancomycin TDM in China, and the implementation of the guideline will be assessed 3 years after its publication.

Conclusion:

This is the first clinical practice guideline that has been developed primarily by pharmacists in China, that strictly follows the IOM new guideline definition and that uses the GRADE approach to rate the quality of evidence and develop recommendations. The guideline will provide the standards of vancomycin TDM in China and help Chinese hospitals conduct vancomycin TDM.

Acknowledgements:

We thank all of the members of the guideline development for their whole-hearted cooperation. Data analysis: ZKY KC YLC SDZ. Manuscript writer: ZKY KC. Study design: ZKY YLC SDZ. Data collection: ZKY KC. Manuscript revision: ZKY KC YLC SDZ. Approved the final version of the manuscript: ZKY KC YLC SDZ.

Competing interest: None

Funding: None

Reference:

1. Cataldo MA, Tacconelli E, Grilli E, et al. Continuous versus intermittent infusion of vancomycin for the treatment of Gram-positive infections: systematic review and meta-analysis. *The Journal of antimicrobial chemotherapy* 2012;67:17-24.
2. Ye ZK, Tang HL, Zhai SD. Benefits of Therapeutic Drug Monitoring of Vancomycin: A Systematic Review and Meta-Analysis. *PloS one* 2013;8.
3. Rybak M, Lomaestro B, Rotschafer JC, et al. Therapeutic monitoring of vancomycin in adult patients: a consensus review of the American Society of Health-System Pharmacists, the Infectious Diseases Society of America, and the Society of Infectious Diseases Pharmacists. *American journal of health-system pharmacy : AJHP : official journal of the American Society of Health-System Pharmacists* 2009;66:82-98.
4. Matsumoto K, Takesue Y, Ohmagari N, et al. Practice guidelines for therapeutic drug monitoring of vancomycin: a consensus review of the Japanese Society of Chemotherapy and the Japanese Society of Therapeutic Drug Monitoring. *Journal of infection and chemotherapy : official journal of the Japan Society of Chemotherapy* 2013;19:365-80.
5. Ye ZK, Li XG, Zhai SD. Therapeutic Drug Monitoring of Vancomycin in China: Current Status and Evaluation. *Chin J Clin Pharmacol* 2013;29:545-8.
6. Ye ZK, Li C, Zhai SD. Guidelines for therapeutic drug monitoring of vancomycin: a systematic review. *PloS one* 2014;9:e99044.
7. Hawser SP, Bouchillon SK, Hoban DJ, et al. Rising incidence of Staphylococcus aureus with reduced susceptibility to vancomycin and susceptibility to antibiotics: a global analysis 2004-2009. *International journal of antimicrobial agents* 2011;37:219-24.
8. Zhuo C, Xu YC, Xiao SN, et al. Glycopeptide minimum inhibitory concentration creep among meticillin-resistant Staphylococcus aureus from 2006-2011 in China. *International journal of antimicrobial agents* 2013;41:578-81.
9. Niki Y, Hanaki H, Matsumoto T, et al. Nationwide surveillance of bacterial respiratory pathogens conducted by the Japanese Society of Chemotherapy in 2008: general view of the pathogens' antibacterial susceptibility. *Journal of infection and chemotherapy : official journal of the Japan Society of Chemotherapy* 2011;17:510-23.
10. Tenover FC, Moellering RC, Jr. The rationale for revising the Clinical and Laboratory Standards Institute vancomycin minimal inhibitory concentration interpretive criteria for Staphylococcus aureus. *Clinical infectious diseases : an official publication of the Infectious Diseases Society of America* 2007;44:1208-15.
11. Institute of Medicine: Clinical Practice Guidelines We Can Trust. Washington, DC: The National Academies Press; 2011.
12. The AGREE Next Steps Consortium. Appraisal of Guidelines for Research and Evaluation. 2009. cited 2013http://www.agreetrust.org/ (accessed 2 June 2013).
13. World Health Organization: WHO Handbook for Guideline Development. 2012. cited 2013http://apps.who.int/iris/bitstream/10665/75146/1/9789241548441_eng.pdf (accessed 2 Nov 2013).
14. Yaolong Chen, Susan Norris, Hao Chen, et al. The Development of a Global Practie Guidelines Registry Platform. 3rd International Society for Evidence-Based Health Care Conference 2014. Taiwan. November 6-9.
15. http://www.guidelines-registry.org/
16. Jaeschke R, etc. Use of GRADE grid to reach decisions on clinical practice guidelines when consensus is elusive. BMJ 2008;337: a744.
17. The RIGHT Group:Essential Reporting Items for Practice Guidelines in Healthcare (RIGHT). 2014. cited 2014 http://www.right-statement.org/ (accessed 5 May 2014).
