# Supplementary material for: Association between the AUC0-24/MIC Ratio of Vancomycin and Its Clinical Effectiveness: A Systematic Review and Meta-Analysis
Source: PLoS One. 2016 Jan 5;11(1):e0146224. doi: 10.1371/journal.pone.0146224 (PMC4701440; doi:10.1371/journal.pone.0146224)
Supplement: S3 File — (DOCX) [file pone.0146224.s004.docx]

| **Excluded articles** | **Reasons** |
| --- | --- |
| de Hoog M, Mouton JW, van den Anker JN. Vancomycin: pharmacokinetics and administration regimens in neonates. Clin Pharmacokinet. 2004;43:417-440. | Did not present sufficient clinical data |
| DeRyke CA, Alexander DP. Optimizing vancomycin dosing through pharmacodynamic assessment targeting area under the concentration-time curve/minimum inhibitory concentration. Hosp Pharm. 2009; 44: 751-765. | Did not compare the clinical outcomes between the high and low AUC0-24/MIC ratio groups |
| Fernández de Gatta Mdel M, Santos Buelga D, Sánchez Navarro A, Dominguez-Gil A, García MJ. Vancomycin dosage optimization in patients with malignant haematological disease by pharmacokinetic/pharmacodynamic analysis. Clin Pharmacokinet. 2009;48:273-80. | Did not compare the clinical outcomes between the high and low AUC0-24/MIC ratio groups |
| Giuliano C1, Haase KK, Hall R. Use of vancomycin pharmacokinetic-pharmacodynamic properties in the treatment of MRSA infections. Expert Rev Anti Infect Ther. 2010;8:95-106. | Did not present sufficient clinical data |
| Inagaki Y, Komatsu M, Yamamoto I, Matsuo S. Clinical significance of pharmacokinetic/pharmacodinamic variables using Monte Carlo simulation in vancomycin treatment of pulmonary methicillin-resistant Staphylococcus aureus infection. Chemotherapy. 2005; 53: 297-301. | Did not compare the clinical outcomes between the high and low AUC0-24/MIC ratio groups |
| Jeffres MN, Isakow W, Doherty JA, McKinnon PS, Ritchie DJ, Micek ST, et al. Predictors of mortality for methicillin-resistant Staphylococcus aureus health-care-associated pneumonia: specific evaluation of vancomycin pharmacokinetic indices. Chest. 2006; 130: 947-955. | Did not compare the clinical outcomes between the high and low AUC0-24/MIC ratio groups |
| Mizokami F, Shibasaki M, Yoshizue Y, Noro T, Mizuno T, Furuta K. Pharmacodynamics of vancomycin in elderly patients aged 75 years or older with methicillin-resistant Staphylococcus aureus hospital-acquired pneumonia. Clin Interv Aging. 2013;8:1015-1021. | Did not compare the clinical outcomes between the high and low AUC0-24/MIC ratio groups |
| Neely MN, Youn G, Jones B, Jelliffe RW, Drusano GL, et al. (2014) Are vancomycin trough concentrations adequate for optimal dosing? Antimicrobial Agents and Chemotherapy 58: 309-316. | Did not present sufficient clinical data |
